# Recombinant polymerase amplification combined with lateral flow strips for the detection of deep-seated *Candida krusei* infections

**DOI:** 10.3389/fcimb.2022.958858

**Published:** 2022-07-29

**Authors:** Mengdi Zhao, Xizhen Wang, Kun Wang, Yuanyuan Li, Yan Wang, Ping Zhou, Lei Wang, Wenjun Zhu

**Affiliations:** ^1^ Department of Materials Science and Engineering, Suzhou University of Science and Technology, Suzhou, China; ^2^ Department of Medicine Laboratory, The Second People’s Hospital of Lianyungang (Cancer Hospital of Lianyungang), Lianyungang, China; ^3^ School of Biotechnology, Jiangsu University of Science and Technology, Zhenjiang, China

**Keywords:** *Candida krusei*, recombinase polymerase amplification, lateral flow strip, ITS2, bases modified

## Abstract

The incidence of *Candida* infections in intensive care units (ICU) has significantly increased in recent years, and these infections have become one of the most serious complications threatening the lives of ICU patients. The proportion of non-*Candida albicans* infections, such as *Candida krusei* and *Candida glabrata* infections, which are resistant to fluconazole, is increasing each year. Early identification of the strains causing *Candida* infections is important for the timely implementation of targeted treatments to save patients’ lives. However, the current methods of direct microscopy, culture, and histopathology, as well as other diagnostic methods, have many shortcomings, such as their low sensitivity and long assay times; therefore, they cannot meet the needs for early clinical diagnosis. Recombinant polymerase amplification (RPA) is a promising isothermal amplification technique that can be performed without sophisticated instruments and equipment, and is suitable for use in resource-poor areas. RPA combined with lateral flow strips (LFS) can be used to rapidly amplify and visualize target genes within 20 min. In this study, RPA-LFS was used to amplify the internal transcribed spacer 2 (ITS2) region of *C. krusei.* The primer-probe design was optimized by introduction of base mismatches (probe modification of five bases) to obtain a specific and sensitive primer-probe combination for the detection of clinical specimens. Thirty-five common clinical pathogens were tested with RPA-LFS to determine the specificity of the detection system. The RPA-LFS system specifically detected *C. krusei* without cross-reaction with other fungi or bacteria. A gradient dilution of the template was tested to explore the lower limit of detection and sensitivity of the assay. The sensitivity was 10 CFU/50 µL per reaction, without interference from genomic DNA of other species. The RPA-LFS and qPCR assays were performed on 189 clinical specimens to evaluate the detection performance of the RPA-LFS system. Seventy-six specimens were identified as *C. krusei*, indicating a detection rate of 40.2%. The results were consistent with those of qPCR and conventional culture methods. The RPA-LFS system established in our study provides a reliable molecular diagnostic method for the detection of *C. krusei*, thus meeting the urgent need for rapid, specific, sensitive, and portable clinical field testing.

## Introduction

Research on *C. krusei* has been dominated by drug resistance studies, and the early and rapid diagnosis of infections remains a clinical challenge (He et al., 2015). Currently, the common clinical diagnostic methods for pathogenic fungi include microscopic observation of morphological features, culture in specific media, serological antigen antibody testing, and histopathological analysis (Zarrinfar et al., 2012; Taghizadeh-Armaki et al., 2017). A variety of clinical specimens are commonly used for testing, such as sputum, urine, stool, nails, hair, pus, cerebrospinal fluid, blood, and bodily fluids (Esmailzadeh et al., 2018; Nourizadeh et al., 2019). Microscopy, culture, and pathology remain the gold standard for diagnosing fungal infections (Graus et al., 2017). However, culture is time-consuming (24–72 hours), does not allow for rapid diagnosis (Stone et al., 2013), is susceptible to environmental contamination, and is dependent on the proficiency of the operator.

Candidaemia is the most common clinically invasive *Candida* infection, and only 30% of patients have positive blood cultures, according to the literature (Quindós, 2014). CHROMagar Candida medium (Nanjing Yiji Biochemical Technology Co., Ltd., Nanjing, China) is commonly used in clinical practice to identify *C. albicans*, *C. krusei*, *C. tropicalis*, and *C. glabrata*, but is limited in the identification of other *Candida* species (Barmar et al., 2021). The diagnostic accuracy of CHROMagar Candida medium is 85.6%, and the misdiagnosis rate is 14.4% (Marr, 2004).

With advances in molecular biology understanding and techniques, various molecular biology methods have been applied to the clinical diagnosis of pathogenic fungal infections to overcome the shortcomings of traditional methods (Yong et al., 2008; Najafzadeh et al., 2021; Kashefi et al., 2021). Protein-based detection methods, such as matrix-assisted laser desorption/ionization time-of-flight mass spectrometry (MALDI-TOF MS, Bruker), and nucleic acid-based diagnostic methods, including DNA fingerprinting, PCR techniques, and DNA hybridization, are currently used as supplements to traditional diagnostic methods, because no standardized guidelines for those methods are available (Alam et al., 2014; Hedayati et al., 2019). Whereas isothermal amplification of nucleic acids amplifies specific DNA or RNA at specific temperatures, traditional PCR techniques require special instruments for automatic temperature control during the denaturation, annealing, and extension steps. Nucleic acid isothermal amplification technology greatly simplifies the instrumentation required for nucleic acid amplification. A constant temperature water bath can be used for the reaction. Moreover, the reaction time is substantially shorter than that of traditional PCR. This method thus meets the clinical needs of simple operation and rapid diagnosis (Jacobsen et al., 2007). The main isothermal amplification techniques reported to date are loop-mediated isothermal amplification (LAMP) (Fallahi et al., 2020), nuclear acid sequence-based amplification (NASBA) (Huang et al., 2019), rolling circle DNA amplification (RCA) (Carinelli et al., 2017), single primer isothermal amplification (SPIA) (Yang et al., 2021), helicase-dependent isothermal DNA amplification (HAD) (Jeong et al., 2009), and strand displaced amplification (SDA) (Zhang et al., 2017), which have been used in clinical practice.

Recombinase polymerase amplification (RPA) is a recently developed thermostatic amplification technology that combines the advantages of the above methods and addresses their shortcomings, thus enabling rapid, on-site, sensitive, and portable diagnosis (Piepenburg et al., 2006). RPA uses recombinase to open the double-stranded DNA and facilitates primer binding to the target fragment, whereas *Bsu* DNA polymerase, which has chain-switching activity, recognizes the 3’ end of the primer and enables stable amplification (Rosser et al., 2015; Dai et al., 2019; Cossio et al., 2021).

In our study, RPA and qPCR primers were designed for the diagnosis of *C. krusei* infections on the basis of the ITS2 sequence. A total of 24 strains of *C. krusei* strains (20 clinical strains and four standard strains) were collected and used as positive control strains. Thirty-five strains of common clinical pathogens were collected as negative control strains. All strains were validated through amplification of the ITS2 sequence. The differences in sensitivity and specificity between the RPA and qPCR techniques for the diagnosis of *C. krusei* infection were investigated and compared.

## Materials and methods

### Ethics statement

The study protocol was approved by the Medical Ethics Committee of the Second People’s Hospital of Lianyungang City (Lianyungang, Jiangsu, China; permit number 2020013), and informed consent was obtained from patients before the collection of clinical specimens.

### Strain acquisition

*C. krusei* ATCC 14243/34135/5258/2159 was purchased from Shanghai Covey Chemical Technology Co., Ltd. (Shanghai, China), and 20 strains of *C. krusei* were isolated from clinical specimens collected from 2020 to 2021. The specificity of the RPA-LFS assay was verified on the basis of the ITS2 sequences (GenBank: AF 246989) of 35 common pathogens stored in our laboratory, including *C. parapsilosis* ATCC 22019, *C. tropicalis* ATCC 20962, *C. albicans* ATCC 10231, *C. auris*, *C. dubliniensis*, *C. glabrata* ATCC 15126, *C. neoformans* ATCC 14116, *A. baumannii* ATCC 19606, *A. fumigatus*, *A. calcoaceticus*, *A. lwoffii*, *A. haemolyticus*, *A. junii*, *E. faecium*, *E. coli *O157, *S. aureus*, *S. capitis*, *S. epidermidis*, *S. haemolyticus*, *S. hominis*, *S. saprophyticus*, *S. warneri*, *S. maltophilia*, *S. pneumonia*, *C. metapsilosis*, *C. orthopsilosis*, *C. gattii*, *K. pneumoniae*, *V. streptococci*, *C. rugosa*, *C. curvatus*, *H. influenzae*, *B. mirabilis*, *E. cloacae*, and *M. tuberculosis* H37Ra. In total, 189 specimens were collected from patients in our hospital with suspected *Candida* infections.

### Genomic DNA extraction

Unless otherwise indicated, all bacterial strains (1 μL of 10^6^ CFU/mL) were boiled at 100°C for 10 min before being used as templates for nucleic acid amplification. For *C. krusei* and other fungi, genomic DNA was extracted and purified from cultures or clinical specimens with a GeneJET Genomic DNA Purification Kit (Tiangen Biotechnology Co., Ltd., Beijing, China) according to the manufacturer’s instructions. Extracted Genomic DNA was quantified with a Qubit 4 fluorometer (Thermo Fisher Scientific), according to the manufacturer’s instructions.

### Primer and probe design and screening

Two pairs of RPA primers based on ITS2 were designed in Primer Premier 5.0 software (Premier Biosoft International, CA, USA). After the sequence of the specific target region was entered, the following primer parameters were set. The product size was set to 80–150 bp. The primer size was set to 30–35 bp, with no more than three consecutive bases with complementary pairing at the 3′ end, a maximum hairpin score of nine, a maximum primer-dimer score of nine, and a maximum poly-X of five. The primers were confirmed with Primer-BLAST on the NCBI website (https://www.ncbi.nlm.nih.gov/tools/primer-blast), which was also used to confirm the species specificity of the sequences of the primers and probes. Forward primer 5, which extended 16 bp upstream of the target, was evaluated for probe and reverse primer performance in Primer Premier 5 software, to avoid the formation of dimers and hairpin structures. Between the probe and reverse primer, we aimed to achieve a probe size of 46−51 bp, a GC content of 30−80%, and a Tm of 55−80°C. The 5’ end of the probe was labeled with FITC, the 3’ end was closed with a C3 spacer, the 5’ end of the forward primer was in the middle of the probe, the first base substitution extending backward was replaced with tetrahydrofuran (THF), and the 5’ end of the reverse primer was labeled with biotin.

### RPA reaction

RPA reactions were performed with a TwistAmp^®^ Liquid DNA Amplification Kit (TwistDx Inc., Maidenhead, UK) according to the manufacturer’s instructions. The 25 µL reaction system contained 12.5 µL of 2× reaction buffer, 2.5 µL of 10× Basic e-mix, 1.25 µL of 20× core mix, 1.2 µL of 10 µM forward primer, 1.2 µL of 10 µM reverse primer, and 4.6 µL of distilled water. A volume of 1.25 µL of 280 mM magnesium acetate and 0.5 µL of template were added to the lid of each reaction tube. After a short centrifugation, the reaction mixture was incubated at 37°C for 20 min. The RPA amplification products were purified with a PCR cleanup kit (Meiji Biotechnology, Shanghai, China) and separated on a 1.5% agarose gel.

### RPA-LFS assay

RPA reactions were as described above, except that each 25 μL reaction contained 1.05 μL of each primer (10 μM), 0.3 μL of probe (10 μM), 1.0 μL of template, and other standard reaction components. Primers and probes were synthesized by Anhui General Biotechnology Co., Ltd. To initiate the reaction, 1.25 μL of magnesium acetate (280 mM) was added, and the reaction mixture was incubated at 37°C for 20 min. Then 5 μL of the amplification product was diluted 20 fold, and the LFS was inserted into 100 μL of the diluted solvent for approximately 2 minutes. The test and control lines were then visually inspected.

### Sensitivity of the RPA-LFS assay

A 10-fold gradient dilution was tested from 10^0^ to 10^6^ CFU/µL (reaction volume of 50 µL containing 1 µL of *C. krusei* inactivation solution). A 10^6^ CFU/µL inactivating solution of another common pathogen (*C. glabrata*) was also prepared for the RPA-LFS reaction.

### Quantitative PCR analysis

The primers and probes are listed in **Table 1**. Primers used for qPCR were targeted to ITS2 of *C. krusei*. The qPCR reaction mixture consisted of 12.5 μL of MonAmp™ TaqMan qPCR mixture (Mona Biologicals Co., Ltd., Suzhou, China), 0.5 μM of forward and reverse primers, 1 μL of genomic DNA, and distilled water to 25 μL. qPCR was performed with a Roche LightCycler 480 qPCR machine, with a program of 95°C for 10 min, followed by 40 cycles of 95°C for 15 s and 55°C for 60 s.

**Table 1:** Primers and probe based on ITS2 of *C. krusei*.

Primers/Probes	Primer Sequences	Size (bp)		Reaction type
*CK*-F1	GCCTTCCGATAACAAAATCAACAGAAAATG		30	RPA
*CK*-R1	ATATTACAACCAGCAGACATGACAGGTAAA		30	
*CK*-F2	AACAAAATCAACAGAAAATGCGGTTTCAGGC		31	
*CK*-R2	CTTCTTTAAGATATTACAACCAGCAGACATG		31	
*CK*-P	FITC-AACAAAATCAACAGGAAACGCGGATTCAGGC[THF]CCTCTAGAGCTCCGAT-C3 spacer		47	RPA-LFS
*CK*-R2B	Biotin-CTTCTTTAAGATATTACAACCAGCAGACATG		31
*CK*-F3	ATTGTTTCGGGTTCTATGTCTGATTGTGAACG		32
*CK*-F4	TGTTTCGGGTTCTATGTCTGATTGTGAACG		30
*CK*-F5	CTATCTTTACGGGAAGTCAACTAGACCAAA		30
*CK*-F6	TAACATTGTTTCGGGTTCTATGTCTGATTG		30
*CK*-F7	GGGTTCTATGTCTGATTGTGAACGTAAACT		30	
F	AAGTTTGGTGTTCCGTTTG		19	qPCR
R	TCTCCTCGGTGCCTCA		16

F, forward primer; R, reverse primer; P, probe.

## Results

### Primer validation screening strategy

**Figure 1:**
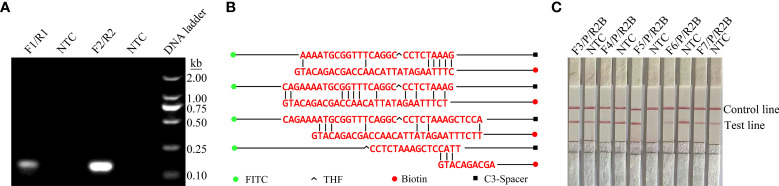
Screening of primer-probe combinations. **(A)** RPA results for two different primer sets against ITS2. The name of each primer set is shown at the top of each lane. NTC indicates the template-free control for the respective primer pair. All reactions were performed at 37°C for 20 min. The images represent the results of three independent experiments. **(B)** Pairwise analysis and sequence modification of the primer-probe set were used to design the ITS2 primers with Primer Premier 5 software, and assess the associated DNA base substitutions of the probes and primers. The DNA strands are shown as horizontal lines, and the matching bases are indicated by vertical lines. Molecular markers are listed in Figure **(B)**. **(C)** Validity of primer-probe sets for the RPA-LFS assay. The name of each primer set is shown at the top of each lane. NTC indicates the no-template control for the respective primer pair. The positions of the test and control lines are shown on the right. All reactions were performed at 37°C for 20 min. The images represent the results of three independent experiments.

ITS2 was selected from the *C. krusei* genome as the target for RPA-LFS detection. Two potential primer pairs were obtained by searching NCBI primer-BLAST for primers specific to the ITS2 sequence (**Table 1**). The primers were initially screened through target gene fragment amplification and a no-template control. The amplification products were electrophoresed on agarose gels to compare the amplification performance of the target gene and primer dimer formation in the no-template control. The primer pair F2/R2 was selected because it showed the best amplification performance and no cross-dimer formation (**Figure 1A**). Candidate probes were obtained by extending the 3’ end of the forward primer F2 by 16 bp. All possible dimers generated by the probe and reverse primer were predicted, and the bases were modified (**Table 1**) until no dimer was formed (**Figure 1B**). Finally, five forward primers were designed, screened, and tested upstream of the probe. The LFS results showed that F3/P/R2B, F4/P/R2B, and F5/P/R2B amplified the target gene fragment efficiently, whereas F6/P/R2B and F7/P/R2B amplified the target less efficiently. The no-template controls of F3/P/R2B, F4/P/R2B, F6/P/R2B, and F7/P/R2B all showed positive results, and only F5/P/R2B met the assay requirements (**Figure 1C**). Therefore, F5/P/R2B was used in subsequent experiments.

### Sensitivity of the RPA-LFS assay

**Figure 2:**
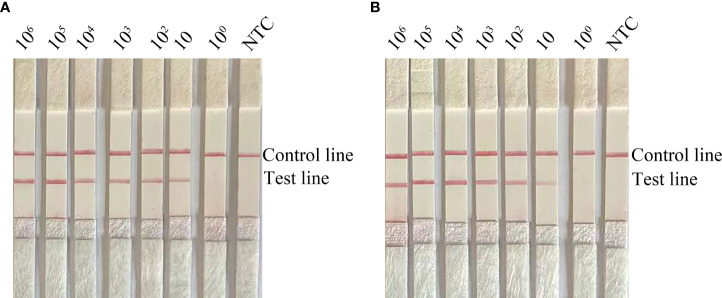
Determination of the limit of detection (LOD) of the *C. krusei* RPA-LFS assay. **(A)** The LOD for the RPA-LFS assay for *C. krusei* was established with the primer-probe set F5/P/R2B on genomic DNA extracted from an inactivated bacterial broth of *C. krusei* at serial dilutions from 10^0^ to 10^6^ CFU for each reaction. The image shows the results of the RPA-LFS. The number of templates is indicated at the top of the bar graph. **(B)** Picture showing the results of the RPA-LFS assay with primer-probe set F5/P/R2B and 10^6^ CFU of *C. glabrata* for interference.

To determine the limit of detection (LOD) of the RPA-LFS system for the detection of *C. krusei*, we tested 10-fold gradient dilutions of *C. krusei* from 10^0^ to 10^6^ CFU/µL (reaction volume: 50 µL, with 1 µL of *C. krusei* genomic DNA added to each reaction). A red band appeared on the test line at 10 CFU/µL and became progressively darker as the concentration of *C. krusei* increased (**Figure 2A**). To test whether other fungal DNA might interfere with the assay, we added 10^6^ CFU/µL of genomic DNA of another common pathogen, *C. glabrata*, to the RPA reaction. The genomic DNA of *C. glabrata* did not interfere with the detection of *C. krusei* (**Figure 2B**). Therefore, we concluded that the LOD for the RPA-LFS system developed in this study was 10 CFU/50 µL per reaction, and genomic DNA from other fungi did not interfere with assay sensitivity.

### Specificity and inclusivity of the RPA-LFS assay

**Figure 3:**
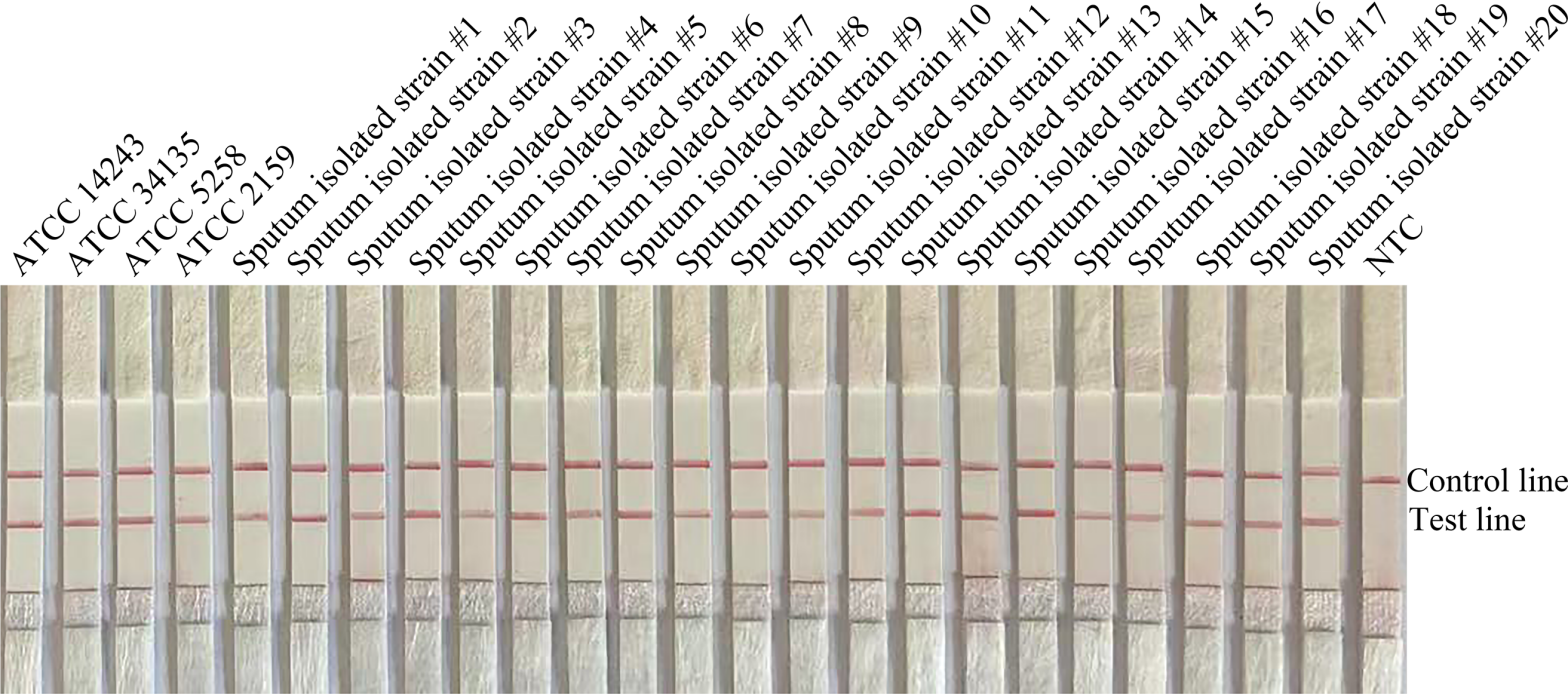
Validation of the specificity of primer pair F5/P/R2B toward *C. krusei*. #1–#20 refer to 20 isolates of *C. krusei* from clinical specimens; NTC indicates no-template control. The positions of the test and control lines are marked on the right side of the image. Reactions were performed at 37°C for 20 min. The image is representative of three independent experiments.

**Figure 4:**
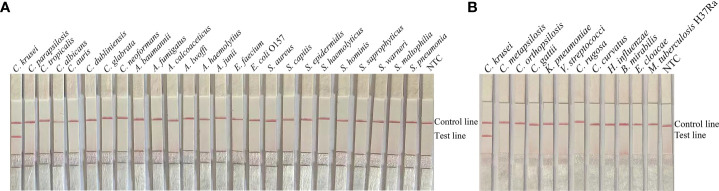
Specificity of F5/P/R2B. *C. krusei* ATCC 14243 was used as a positive control for other pathogenic bacteria tested.**(A, B)** Species names are indicated at the top of each strip. NTC indicates the no-template control specimen. The positions of the test and control lines are indicated on the right side of the image. Reactions were performed at 37°C for 20 min. The image is representative of the results of three independent experiments.

To confirm the inclusivity and specificity of F5/P/R2B, we performed RPA-LFS on four reference strains (*C. krusei* ATCC 14243/34135/5258/2159), 20 clinical isolates (sputum isolated strains no. 1 to 20), and other common clinical pathogens (**Table 2**). The four reference strains and 20 clinical isolates showed positive results (**Figure 3**), whereas all other pathogenic cultures showed negative results (**Figure 4**). Thus, the primer-probe set showed good inclusion and specificity for *C. krusei*, effectively detecting this species without cross-reaction with other pathogens.

**Table 2:** Yeast and bacterial strains used in this study.

Species	Source	Strain designation
*C. krusei*	Reference strain	ATCC 14243
*C. krusei*	Reference strain	ATCC 34135
*C. krusei*	Reference strain	ATCC 5258
*C. krusei*	Reference strain	ATCC 2159
*C. krusei*	Sputum isolated strain	#1–#20
*C. parapsilosis*	Reference strain	ATCC 22019
*C. tropicalis*	Reference strain	ATCC 20962
*C. albicans*	Reference strain	ATCC 10231
*C. auris*	Sputum isolated strain	N/A
*C. dubliniensis*	Sputum isolated strain	N/A
*C. glabrata*	Reference strain	ATCC 15126
*C. neoformans*	Reference strain	ATCC 14116
*A. baumannii*	Reference strain	ATCC 19606
*A. fumigatus*	Sputum isolated strain	N/A
*A. calcoaceticus*	Sputum isolated strain	N/A
*A. lwoffii*	Sputum isolated strain	N/A
*A. haemolyticus*	Sputum isolated strain	N/A
*A. junii*	Sputum isolated strain	N/A
*E. faecium*	Sputum isolated strain	N/A
*E. coli* O157	Sputum isolated strain	N/A
*S. aureus*	Sputum isolated strain	N/A
*S. capitis*	Sputum isolated strain	N/A
*S. epidermidis*	Sputum isolated strain	N/A
*S. haemolyticus*	Sputum isolated strain	N/A
*S. hominis*	Sputum isolated strain	N/A
*S. saprophyticus*	Sputum isolated strain	N/A
*S. warneri*	Sputum isolated strain	N/A
*S. maltophilia*	Sputum isolated strain	N/A
*S. pneumonia*	Sputum isolated strain	N/A
*C. metapsilosis*	Sputum isolated strain	N/A
*C. orthopsilosis*	Sputum isolated strain	N/A
*C. gattii*	Sputum isolated strain	N/A
*K. pneumoniae*	Sputum isolated strain	N/A
*V. streptococci*	Sputum isolated strain	N/A
*C. rugosa*	Sputum isolated strain	N/A
*C. curvatus*	Sputum isolated strain	N/A
*H. influenzae*	Sputum isolated strain	N/A
*B. mirabilis*	Sputum isolated strain	N/A
*E. cloacae*	Sputum isolated strain	N/A
*M. tuberculosis* H37Ra	Sputum isolated strain	N/A

ATCC, American Type Culture Collection (Manassas, VA, USA). N/A, Not Applicable.

### Clinical specimen testing

To verify the practical application of RPA-LFS, we collected 189 clinical specimens for RPA-LFS and qPCR assays. The results are shown in **Table 3**. Seventy-six specimens contained *C. krusei*, and had a detection rate of 40.2%, whereas 113 tests indicated non-*C. krusei*, in agreement with the detection results with qPCR and traditional culture methods. Neither method produced false positive or false negative results. RPA-LFS had the same accuracy as qPCR, and the results were consistent with those of the traditional culture method.

**Table 3:** Detection of the RPA-LFS system and qPCR for *C. krusei*.

RPA-LFS assay				
		Positive	Negative	Total
qPCR	Positive	76	0	76
	Negative	0	113	113
Total		76	113	189

## Discussion

*Candida*, *Aspergillus*, and *Cryptococcus* are the three most common clinically invasive fungi. Among these, *Candida* species are the most common clinically opportunistic fungi, with the highest reported carriage rate for the digestive tract (50%), vagina (20%–30%), skin surface (2%), and pharynx (1–4%). *Candida* can cause diseases when the body’s immunity is low (Zarrinfar et al., 2016). Two main factors influence susceptibility to *Candida* infections in humans: (1) the human body itself, including factors such as organ transplantation, low neutrophil counts, or impaired cellular immunity, and (2) external factors, such as broad-spectrum antibiotics and surgical procedures resulting in nosocomial infections. *Candida* infections have been found to affect 80% of patients hospitalized in the ICU for more than 1 week; moreover, 10% of these patients may develop invasive infections, thus threatening their prognosis (Arendrup et al., 2011; Gökahmetoğlu et al., 2016). At least 17 species of *Candida* cause human disease, among which *C. albicans* causes the most common infections (Minooeianhaghighi et al., 2019). However, in recent years, with organ transplantation, and the use of immunosuppressive agents, broad-spectrum antibiotics, and the increasing application of glucocorticoids, the spectrum of common clinical *Candida* species has changed. *C. krusei*, *C. glabrata*, *C. tropicalis*, and *C. parapsilosis* have increasingly caused non-*Candida albicans* infections, accounting for 35%–65% of cases (Krcmery and Barnes, 2002; Mohammadi et al., 2013; Guinea et al., 2014). *C. krusei* is a common clinical opportunistic pathogenic that can cause limited or systemic infections in humans, and has attracted research attention because of its natural resistance to fluconazole and its increasing prevalence. *C. krusei* results in a high mortality rate after infection, and early and rapid diagnosis is important for patient prognosis.

The traditional diagnostic methods, based on the morphological and physiological characteristics of *C. krusei*, are time-consuming and complicated to perform, thus often delaying patient treatment. Automatic or manual rapid diagnostic kits have become available for clinical applications, thereby overcoming several shortcomings of traditional diagnostic methods. With the development of molecular biology diagnostic technologies, related diagnostic techniques have been applied in clinical settings for early and rapid diagnosis of pathogenic fungi. In recent years, many nucleic acid amplification-based methods have been reported for the diagnosis of infections with common clinical *Candida* species, including *C. krusei*. Brito et al. (2009) initially reported the use of ITS primers to amplify *Candida* DNA and identify the species class according to the length of electrophoretic bands. The authors found that the ITS3 and ITS4 primer pairs amplified fragments of similar length (at 331 bp) for *C. tropicalis* and *C. krusei*, thus failing to distinguish these species. Therefore, the ITS2 primer pair, which allowed for good differentiation between *C. krusei* and other *Candida* species, was chosen to perform the initial strain validation test.

Khlif et al. (2009) have used nested PCR for the diagnosis of common clinical *Candida* infections and have performed two PCR reactions, thereby improving the sensitivity and specificity. However, that method is more complicated than RPA and requires an expensive PCR instrument to perform the reactions.

A qPCR assay has been described by Decat et al. (2013); however, in that study, the most closely related strains of *C. krusei* were not included as controls. The RPA test herein used primers targeting the ITS2 sequence, and was able to achieve specific amplification within 2.5 hours and provide advantages over qPCR. A comparison of the sensitivity, specificity, practicality, and speed of the RPA and qPCR techniques, indicated that both RPA and qPCR techniques had 100% specificity in the diagnosis of *C. krusei* infection. However, RPA has the benefits of being a thermostatic amplification technique that does not require temperature changes to achieve specific amplification of DNA and can be combined with LFS to meet visualization needs. Although RPA does not require high experimental conditions, non-specific amplification due to aerosol contamination can be problematic. This drawback can be prevented through strict spatial partitioning to isolate the areas in which reaction and detection are performed. qPCR requires specific fluorescence PCR instruments and is expensive; moreover, it requires 2.5 hours to complete, whereas RPA can be completed in 20 min (Wu et al., 2017; Tian et al., 2018; Wang et al., 2021).

Overall, the RPA technique was easy to perform, the reaction results were readily determined, the time required for the reaction was short, and no specialized instrumentation was necessary. Thus, this method may enable early diagnosis in resource-poor areas. Many technologies have been reported, such as those used to detect *Listeria monocytogenes*, *C. albicans*, or *C. neoformans* (Wang et al., 2019; Wang et al., 2021; Wang et al., 2022). However, the traditional diagnostic methods for pathogenic fungi, which are commonly used in clinical practice, are time-consuming, prone to false-positive and false-negative results, and susceptible to researchers. Molecular biology diagnostic techniques have been gradually applied in clinical practice because of their time-consuming and high specificity. In this study, we diagnosed fluconazole-resistant *C. krusei* by using the RPA-LFS technique, which is rapid and specific, and therefore may aid in the early diagnosis of *C. krusei* infections in clinical settings.

## Data availability statement

The original contributions presented in the study are included in the article/supplementary material. Further inquiries can be directed to the corresponding authors.

## Author contributions

MDZ, WJZ, and LW designed the experiments and wrote the manuscript. YYL and YW collected the clinical samples. KW and XZW performed the experiments. MDZ and PZ analyzed the data. All authors reviewed and approved the final version of the manuscript.

## Funding

This study was supported by grants from the Researching Phase Transition of Confined System by Theory and Simulation (grant no.332114411), the Lianyungang Science and Technology Bureau, Municipal Science and Technology Plan (Social Development) (grant no. SF2140), the Lianyungang City Health Science and Technology Project (grant no. 202122), and the Jiangsu University Clinical Medicine Science and Technology Development Fund (grant no. JLY2021088).

## Acknowledgments

We thank International Science Editing (http://www.internationalscienceediting.com) for editing this manuscript.

## Conflict of interest

The authors declare that the research was conducted in the absence of any commercial or financial relationships that could be construed as a potential conflict of interest.

## Publisher’s note

All claims expressed in this article are solely those of the authors and do not necessarily represent those of their affiliated organizations, or those of the publisher, the editors and the reviewers. Any product that may be evaluated in this article, or claim that may be made by its manufacturer, is not guaranteed or endorsed by the publisher.

## References

[B1] Alam M. Z. Alam Q. Jiman-Fatani A. Kamal M. A. Abuzenadah A. M. Chaudhary A. G.. (2014). *Candida* identification: a journey from conventional to molecular methods in medical mycology. World J. Microbiol. Biotechnol. 30 (5), 1437–1451. doi: 10.1007/s11274-013-1574-z 24379160

[B2] Arendrup M. C. Sulim S. Holm A. Nielsen L. Nielsen S. D. Knudsen J. D.. (2011). Diagnostic issues, clinical characteristics, and outcomes for patients with fungemia. J. Clin. Microbiol. 49 (9), 3300–3308. doi: 10.1128/jcm.00179-11 21715585 PMC3165619

[B3] Barmar P. Zarrinfar H. Jarahi L. Fata A. (2021). Comparison of candida species in patients with *Candida* vulvovaginitis in torbat-e jam and its relationship with diabetes. Iranian J. Obstetrics Gynecol Infertility 23 (11), 60–67. doi: 10.22038/IJOGI.2021.17621Gynecology, infertility

[B4] Brito E. H. Brilhante R. S. Cordeiro R. A. Sidrim J. J. Fontenelle R. O. Melo L. M.. (2009). PCR-AGE, automated and manual methods to identify *Candida* strains from veterinary sources: a comparative approach. Veterinary Microbiol. 139 (3-4), 318–322. doi: 10.1016/j.vetmic.2009.06.031 19592181

[B5] Carinelli S. Kühnemund M. Nilsson M. Pividori M. I. (2017). Yoctomole electrochemical genosensing of Ebola virus cDNA by rolling circle and circle to circle amplification. Biosensors Bioelectronics 93, 65–71. doi: 10.1016/j.bios.2016.09.099 27838201

[B6] Cossio A. Jojoa J. Castro M. D. M. Castillo R. M. Osorio L. Shelite T. R.. (2021). Diagnostic performance of a recombinant polymerase amplification test-lateral flow (RPA-LF) for cutaneous leishmaniasis in an endemic setting of Colombia. PloS Negl. Trop. Dis. 15 (4), e0009291. doi: 10.1371/journal.pntd.0009291 33909619 PMC8081229

[B7] Dai T. Yang X. Hu T. Jiao B. Xu Y. Zheng X.. (2019). Comparative evaluation of a novel recombinase polymerase amplification-lateral flow dipstick (RPA-LFD) assay, LAMP, conventional PCR, and leaf-disc baiting methods for detection of phytophthora sojae. Front. Microbiol. 10. doi: 10.3389/fmicb.2019.01884 PMC6696978 31447827

[B8] Decat E. Van Mechelen E. Saerens B. Vermeulen S. J. Boekhout T. De Blaiser S.. (2013). Rapid and accurate identification of isolates of *Candida* species by melting peak and melting curve analysis of the internally transcribed spacer region 2 fragment (ITS2-MCA). Res. Microbiol. 164 (2), 110–117. doi: 10.1016/j.resmic.2012.10.017 23142490

[B9] Esmailzadeh A. Zarrinfar H. Fata A. Sen T. (2018). High prevalence of candiduria due to non-albicans *Candida* species among diabetic patients: A matter of concern? J. Clin. Lab. Anal. 32 (4), e22343. doi: 10.1002/jcla.22343 29076587 PMC6817075

[B10] Fallahi S. Babaei M. Rostami A. Mirahmadi H. Arab-Mazar Z. Sepahvand A. (2020). Diagnosis of *Candida* albicans: conventional diagnostic methods compared to the loop-mediated isothermal amplification (LAMP) assay. Arch. Microbiol. 202 (2), 275–282. doi: 10.1007/s00203-019-01736-7 31641798

[B11] Gökahmetoğlu G. Mutlu Sarıgüzel F. Koç A. N. Behret O. Gökahmetoğlu S. Atalay M. A.. (2016). [Determination of *Candida* colonization and *Candida* score in patients in anesthesia intensive care unit]. Mikrobiyol Bulteni 50 (3), 438–448. doi: 10.5578/mb.27685 27525399

[B12] Graus M. S. Neumann A. K. Timlin J. A. (2017). Hyperspectral fluorescence microscopy detects autofluorescent factors that can be exploited as a diagnostic method for *Candida* species differentiation. J. Biomed. Optics 22 (1), 16002. doi: 10.1117/1.Jbo.22.1.016002 PMC5216876 28056142

[B13] Guinea J. Zaragoza Ó Escribano P. Martín-Mazuelos E. Pemán J. Sánchez-Reus F.. (2014). Molecular identification and antifungal susceptibility of yeast isolates causing fungemia collected in a population-based study in Spain in 2010 and 2011. Antimicrob Agents Chemother 58 (3), 1529–1537. doi: 10.1128/aac.02155-13 24366741 PMC3957835

[B14] Hedayati M. T. Taghizadeh-Armaki M. Zarrinfar H. Hoseinnejad A. Ansari S. Abastabar M.. (2019). Discrimination of aspergillus flavus from aspergillus oryzae by matrix-assisted laser desorption/ionisation time-of-flight (MALDI-TOF) mass spectrometry. Mycoses 62 (12), 1182–1188. doi: 10.1111/myc.13010 31556203

[B15] He X. Zhao M. Chen J. Wu R. Zhang J. Cui R.. (2015). Overexpression of both ERG11 and ABC2 genes might be responsible for itraconazole resistance in clinical isolates of *Candida* krusei. PloS One 10 (8), e0136185. doi: 10.1371/journal.pone.0136185 26308936 PMC4550294

[B16] Huang C. Huang P. T. Yao J. Y. Li Z. W. Weng L. B. Guo X. G. (2019). Pooled analysis of nuclear acid sequence-based amplification for rapid diagnosis of mycoplasma pneumoniae infection. J. Clin. Lab. Anal. 33 (5), e22879. doi: 10.1002/jcla.22879 30843291 PMC6595323

[B17] Jacobsen M. D. Gow N. A. Maiden M. C. Shaw D. J. Odds F. C. (2007). Strain typing and determination of population structure of *Candida* krusei by multilocus sequence typing. J. Clin. Microbiol. 45 (2), 317–323. doi: 10.1128/jcm.01549-06 17122025 PMC1829042

[B18] Jeong Y. J. Park K. Kim D. E. (2009). Isothermal DNA amplification *in vitro*: the helicase-dependent amplification system. Cell. Mol. Life sciences: CMLS 66 (20), 3325–3336. doi: 10.1007/s00018-009-0094-3 PMC11115679 19629390

[B19] Kashefi E. Seyedi S. Zarrinfar H. Fata A. Mehrad-Majd H. Najafzadeh M. (2021). Molecular identification of *Candida* species in bronchoalveolar lavage specimens of hospitalized children with pulmonary disorders. J. Babol Univ. Of Med. Sci. 23 (1), 331–336. doi: 10.22088/jbums.23.1.331

[B20] Khlif M. Mary C. Sellami H. Sellami A. Dumon H. Ayadi A.. (2009). Evaluation of nested and real-time PCR assays in the diagnosis of candidaemia. Clin. Microbiol. infection 15 (7), 656–661. doi: 10.1111/j.1469-0691.2009.02762.x 19438623

[B21] Krcmery V. Barnes A. J. (2002). Non-albicans *Candida* spp. causing fungaemia: Pathogenicity and antifungal resistance. J. Hosp. Infection 50 (4), 243–260. doi: 10.1053/jhin.2001.1151 12014897

[B22] Marr K. A. (2004). Invasive *Candida* infections: The changing epidemiology. Oncol. (Williston Park NY) 18 (14 Suppl 13), 9–14. 15682589

[B23] Minooeianhaghighi M. H. Sehatpour M. Zarrinfar H. Sen T. (2019). Recurrent vulvovaginal candidiasis: The causative agents, clinical signs and susceptibility to fluconazole in gonabad city, the northeast of Iran. Curr. Women s Health Rev. 15, 46–51. doi: 10.2174/1573404815666191104142813

[B24] Mohammadi R. Mirhendi H. Rezaei-Matehkolaei A. Ghahri M. Shidfar M. R. Jalalizand N.. (2013). Molecular identification and distribution profile of *Candida* species isolated from Iranian patients. Med. Mycol 51 (6), 657–663. doi: 10.3109/13693786.2013.770603 23470036

[B25] Najafzadeh M. J. Dolatabadi S. Zarrinfar H. Houbraken J. (2021). Molecular diversity of aspergilli in two Iranian hospitals. Mycopathologia 186 (4), 519–533. doi: 10.1007/s11046-021-00563-z 34052941

[B26] Nourizadeh N. Adabizadeh A. Zarrinfar H. Majidi M. Jafarian A. H. Najafzadeh M. J. (2019). Fungal biofilms in sinonasal polyposis: The role of fungal agents is notable? J. Oral. Maxillofac. Surgery Med Pathol. 31 (4), 295–298. doi: 10.1016/j.ajoms.2019.01.007

[B27] Piepenburg O. Williams C. H. Stemple D. L. Armes N. A. (2006). DNA Detection using recombination proteins. PloS Biol. 4 (7), e204. doi: 10.1371/journal.pbio.0040204 16756388 PMC1475771

[B28] Quindós G. (2014). Epidemiology of candidaemia and invasive candidiasis. a changing face. Rev. Iberoamericana Micolog 31 (1), 42–48. doi: 10.1016/j.riam.2013.10.001 24270071

[B29] Rosser A. Rollinson D. Forrest M. Webster B. L. J. P. (2015). Isothermal recombinase polymerase amplification (RPA) of schistosoma haematobium DNA and oligochromatographic lateral flow detection. Parasites Vectors 8 (1), 446. doi: 10.1186/s13071-015-1055-3 26338510 PMC4559068

[B30] Stone N. R. Gorton R. L. Barker K. Ramnarain P. Kibbler C. C. (2013). Evaluation of PNA-FISH yeast traffic light for rapid identification of yeast directly from positive blood cultures and assessment of clinical impact. J. Clin. Microbiol. 51 (4), 1301–1302. doi: 10.1128/jcm.00028-13 23390280 PMC3666762

[B31] Taghizadeh-Armaki M. Hedayati M. T. Moqarabzadeh V. Ansari S. Mahdavi Omran S. Zarrinfar H.. (2017). Effect of involved aspergillus species on galactomannan in bronchoalveolar lavage of patients with invasive aspergillosis. J. Med. Microbiol. 66 (7), 898–904. doi: 10.1099/jmm.0.000512 28693685 PMC5737110

[B32] Tian A. L. Elsheikha H. M. Zhou D. H. Wu Y. D. Chen M. X. Wang M.. (2018). A novel recombinase polymerase amplification (RPA) assay for the rapid isothermal detection of neospora caninum in aborted bovine fetuses. Veterinary Parasitol. 258, 24–29. doi: 10.1016/j.vetpar.2018.06.004 30105974

[B33] Wang F. Ge D. Wang L. Li N. Chen H. Zhang Z.. (2021). Rapid and sensitive recombinase polymerase amplification combined with lateral flow strips for detecting *Candida* albicans. Analytical Biochem. 633, 114428. doi: 10.1016/j.ab.2021.114428 34678249

[B34] Wang L. Wang Y. Wang F. Zhao M. Gao X. Chen H.. (2022). Development and application of rapid clinical visualization molecular diagnostic technology for *Cryptococcus neoformans*/*C. gattii* based on recombinase polymerase amplification combined with a lateral flow strip. Front. Cell Infect. Microbiol. 11. doi: 10.3389/fcimb.2021.803798 PMC8790172 35096653

[B35] Wang L. Zhao P. Si X. Li J. Dai X. Zhang K.. (2019). Rapid and specific detection of listeria monocytogenes with an isothermal amplification and lateral flow strip combined method that eliminates false-positive signals from primer-dimers. Front. Microbiol. 10. doi: 10.3389/fmicb.2019.02959 PMC7025549 32117075

[B36] Wu Y. D. Xu M. J. Wang Q. Q. Zhou C. X. Wang M. Zhu X. Q.. (2017). Recombinase polymerase amplification (RPA) combined with lateral flow (LF) strip for detection of toxoplasma gondii in the environment. Veterinary Parasitol. 243, 199–203. doi: 10.1016/j.vetpar.2017.06.026 28807294

[B37] Yang Q. Guo W. Liu Y. Zhang Y. Ming R. Yuan Y.. (2021). Novel single primer isothermal amplification method for the visual detection of vibrio parahaemolyticus. Food Analytical Methods 14 (10), 1995–2002. doi: 10.1007/s12161-021-02033-0

[B38] Yong P. V. Chong P. P. Lau L. Y. Yeoh R. S. Jamal F. (2008). Molecular identification of *Candida* orthopsilosis isolated from blood culture. Mycopathologia 165 (2), 81–87. doi: 10.1007/s11046-007-9086-8 18266075

[B39] Zarrinfar H. Kaboli S. Dolatabadi S. Mohammadi R. (2016). Rapid detection of *Candida* species in bronchoalveolar lavage fluid from patients with pulmonary symptoms. Braz. J. Microbiol. 47 (1), 172–176. doi: 10.1016/j.bjm.2015.02.001 26887241 PMC4822774

[B40] Zarrinfar H. Saber S. Kordbacheh P. Makimura K. Fata A. Geramishoar M.. (2012). Mycological microscopic and culture examination of 400 bronchoalveolar lavage (BAL) samples. Iran J. Public Health 41 (7), 70–76. PMC3469012 23113213

[B41] Zhang M. Li R. Ling L. (2017). Homogenous assay for protein detection based on proximity DNA hybridization and isothermal circular strand displacement amplification reaction. Analytical Bioanalytical Chem. 409 (16), 4079–4085. doi: 10.1007/s00216-017-0356-0 28424856

